# Prospective Mapping of Transcriptional Changes Associated with Lipid and Carotenoid Production in *Rhodotorula glutinis* Using Different Feeding Approaches

**DOI:** 10.3390/biology15010060

**Published:** 2025-12-29

**Authors:** Nora Elfeky, Yongheng Yang, Guoping Zhu, Yongming Bao

**Affiliations:** 1 Anhui Provincial Key Laboratory of Molecular Enzymology and Mechanism of Major Metabolic Diseases, College of Life Sciences, Anhui Normal University, Wuhu 241002, China; nora.elfeky2021@science.menofia.edu.eg; 2 School of Biomedical Engineering, Dalian University of Technology, Dalian 116024, China; 3 Botany Department, Faculty of Science, Menoufia University, Shebin El-Koom 32511, Egypt; 4 School of Food and Environmental Engineering, Dalian University of Technology, Panjin 12422, China; y-yongheng@zust.edu.cn

**Keywords:** *Rhodotorula glutinis*, RNA transcriptomic analysis, lipids, carotenoids, FAS, carotene hydroxylase, phytoene synthase, bifunctional lycopene cyclase/phytoene synthase

## Abstract

The oleaginous yeast *Rhodotorula glutinis* is a promising industrial host for the simultaneous production of lipids and carotenoids, yet the transcriptional regulation governing carbon flux toward these metabolites is poorly understood. As a foundational step, we performed a comparative transcriptomic analysis on bioreactor cultures under optimized fed-batch conditions with varying carbon-to-nitrogen (C/N) ratios and metal supplementation, comparing a nutrient-replete control (C) with conditions favoring high lipid (HLP) or high carotenoid (HCP) production. This study was designed as a preliminary, in-depth case study using single, well-controlled bioreactor runs per condition, with the goal of generating a comprehensive transcriptional map to identify key candidate genes for future validation. The data delineates two distinct presumptive metabolic strategies. The HLP regime was associated with broad transcriptional downregulation, channeling carbon toward lipogenesis via specific upregulation of the fatty acid synthase complex (FAS1/2, Log_2_FC(HLP/HCP) > 2.99) and concerted suppression of β-oxidation genes (Log_2_FC < −9.70). Conversely, the HCP condition was characterized by significant upregulation of NADPH-supplying pathways, including the pentose phosphate pathway (e.g., rpiA, Log_2_FC(HCP/C) = 11.39) and an NADP^+^-dependent glyceraldehyde-3-phosphate dehydrogenase (gapN, Log_2_FC(HCP/C) = 12.24). Notably, a putative beta-carotene hydroxylase (CrtZ) was uniquely sustained in the HCP condition (Log_2_FC(HLP/HCP) = −10.65), strongly correlating with torularhodin accumulation and suggesting its novel role in torulene hydroxylation. This exploratory study reveals prospective transcriptional determinants of carbon partitioning in *R. glutinis* and provides a prioritized genetic blueprint for future hypothesis-driven research with full biological replication.

## Simple Summary

The industrial potential of the yeast *Rhodotorula glutinis* to produce valuable lipids and carotenoids is hampered by an unclear understanding of how it directs carbon toward one product or the other. As a foundational step to decipher this critical regulatory switch, we performed an in-depth comparative transcriptomic case study using optimized bioreactor conditions. Our analysis reveals two distinct and opposing presumptive metabolic strategies: lipid maximization is associated with a broad genetic downregulation and specific upregulation of fat synthesis, while carotenoid production correlates with a boosted cellular NADPH supply and the unique retention of a key enzyme (CrtZ), potentially linked to torularhodin synthesis. This exploratory work provides a foundational genetic map and prioritizes precise molecular targets for future hypothesis-driven engineering of specialized, high-efficiency strains for sustainable bio-oil or high-value pigment production, pending validation with biological replication.

## 1. Introduction

*Rhodotorula glutinis* (*R. glutinis*) is an oleaginous yeast renowned for its simultaneous production of lipids, suitable for high-quality biodiesel, and carotenoid pigments, such as torulene and torularhodin, which possess significant industrial and antioxidant value [1,2,3]. The fatty acids profile of its lipids, which is rich in oleic (18:1), palmitic (16:0), and linoleic acid (18:2), meet key biodiesel criteria [2,4]. Furthermore, its unique carotenoids, torulene and torularhodin, are of growing interest [5].

A critical, unresolved question in the biology of *R. glutinis* is the metabolic relationship between lipid and carotenoid biosynthesis. These pathways share acetyl-CoA as a common precursor, yet reported interactions are contradictory, showing both positive and negative correlations under various culture conditions [6,7,8]. Furthermore, while the lipid metabolism pathway is well-characterized in oleaginous yeasts [9], the carotenoid pathway in *R. glutinis* remains insufficiently understood, with key enzymes for the conversion of torulene to torularhodin still unidentified [10].

Our previous work demonstrated that manipulating culture conditions, specifically metal supplementation, carbon to sulfur (C/S), and carbon to nitrogen (C/N) ratios, can shift metabolism to preferentially maximize either carotenoid or lipid production [2,11]. However, the underlying cellular and molecular mechanisms driving this reversible relationship are unknown. Although transcriptomics has been used to study stress resistance in *R. glutinis* [12] and lipid metabolism in related species [13], a comparative analysis of the transcriptional regulation governing the split between lipid and carotenoid pathways in *R. glutinis* is lacking.

Therefore, we performed a deep, comparative transcriptomic case study on meticulously controlled single bioreactor cultivations under C, HLP, and HCP regimes. The primary objective was to generate a comprehensive, foundational dataset and formulate specific, testable hypotheses about the transcriptional wiring of lipid and carotenoid metabolism in *R. glutinis* to pinpoint the crucial regulatory enzymes responsible for this metabolic shift, thereby providing a genetic foundation for the targeted engineering of these pathways.

## 2. Materials and Methods

### 2.1. Fed-Batch Fermentation of R. glutinis

Three different cultivation media, with feeding solutions, were used for the transcriptomic analysis of *Rhodotorula glutinis* (*R. glutinis*) (AS 2.703) obtained from the China General Microbiological Culture Collection Center (CGMCC, Beijing, China). The media and feeding solution composition is provided in Table 1. The first experimental group exhibits a low C/N ratio, which was regarded as the control group (C). The second experimental group reflects the optimal combination of medium composition and feeding solution for achieving high lipid production (HLP) [2]. The third group is the optimal medium for achieving the highest carotenoid synthesis by *R. glutinis* (HCP) [11]. Following medium preparation, it was introduced into a 5 L bioreactor equipped with a dissolved oxygen (DO) electrode and a pH electrode. Subsequently, it underwent sterilization at a temperature of 121 °C and a pressure of 1.5 atm for a duration of 20 min. Following the cooling process, the 5 L bioreactor was injected with the seed culture. Throughout the entire experiment, the temperature, pH, and agitation were automatically set at 28 °C, 5, and 400 rpm, respectively. After a duration of 96 h, the samples were collected for the purpose of extracting RNA and conducting the analytical analysis.

### 2.2. Detection of Lipid Bodies, Dry Cell Weight (DCW) and Reducing Sugar in the Culture Media

Detection of lipid bodies, DCW, as well as residual sugar in the medium was performed as described previously by Elfeky et al. [11]. For lipid visualization, cells from a 100 µL sample were washed and resuspended in 10 mM phosphate buffer (pH 7, containing 0.15 M potassium hydroxide). They were then stained with 10 µL of a Nile red solution (1 mg/mL in acetone, stored in darkness at 4 °C). After a 5 min incubation in the dark, cells were examined under an Olympus IX71 fluorescence microscope fitted with a blue fluorescence cube and camera (Olympus, Tokyo, Japan) to observe cell morphology and the golden fluorescent lipid bodies within [14]. DCW was measured by centrifuging a 5 mL sample at 10,000 rpm for 10 min. The cell pellet was washed twice with sterilized distilled water, freeze-dried, and weighed [15]. The resulting supernatant was used to determine residual sugar concentration via the 3,5-dinitrosalicylic acid method [16].

### 2.3. Total Lipids (TL) Detection and GC Analysis of Fatty Acids Methyl Esters

Total lipids in the yeast cells were detected using the sulfo-phospho-vanillin method [17] as described by Elfeky et al. [11]. Briefly, the lipid content was determined from a 200 µL yeast culture sample mixed with 2 mL of concentrated H_2_SO_4_ and 100 µL distilled water. After heating at 100 °C for 10 min and cooling on ice for 5 min, 5 mL of freshly prepared phospho-vanillin reagent (PVR) was added. The mixture was incubated at 37 °C with shaking at 200 rpm for 15 min, followed by a 40 min incubation in darkness. Absorbance was then measured at 530 nm. A calibration curve was generated by subjecting known concentrations of olive oil to the same procedure. For determining the fatty acids composition, transesterification of the samples was carried out according to Van Wychen et al. [18], as described by Elfeky et al. [2]. Freeze-dried cells from a 10 mL yeast suspension were placed into vials preheated to 85 °C. A mixture of 200 µL chloroform:methanol (2:1 *v*/*v*) and 300 µL of 0.6 M HCl in methanol was added to each vial. The sealed vials were vortexed thoroughly and heated again at 85 °C for 60 min. After cooling for 15 min at room temperature, 1 mL of hexane was added, and the vials were vortexed to mix. Subsequently, 1 mL of a 0.1% NaOH solution was added to wash the acid. The mixture was centrifuged at 2000 rpm for 5 min to separate the phases. The hexane (upper) layer, containing the fatty acid methyl esters (FAMEs), was carefully transferred to a new GC vial for analysis. FAME analysis was performed using an Agilent 7890A gas chromatograph equipped with an autosampler and a flame ionization detector (FID) (Agilent Technologies, Santa Clara, CA, USA). Separation was achieved using an HP-FFAP capillary column (25 m length, 0.2 mm internal diameter, 0.33 µm film thickness) (Agilent Technologies, Santa Clara, CA, USA). The injector and detector temperatures were maintained at 240 °C. The column temperature was held at 180 °C for 2 min, then increased to 240 °C at a rate of 7 °C/min and held for the final 2 min. Individual fatty acids were identified by comparing their retention times to known standards and were quantified as a percentage of the total FAME content.

### 2.4. Extraction, Quantification and Identification of Total Carotenoids

Extraction, identification, and quantification of carotenoids were performed as described by Elfeky et al. [11]. Carotenoid extraction was performed using freeze-dried biomass from a 10 mL culture. The cells were first hydrolyzed by boiling with 1 M HCl for 5 min, then centrifuged and washed to a neutral pH. The hydrolyzed pellet was subjected to a sequential solvent extraction: 1 mL of acetone was added first, followed by 0.5 mL of ethyl acetate and 0.5 mL of petroleum ether. This gradual addition was found to improve extraction yield compared to adding a pre-mixed solvent. The mixture was then washed with 5 mL of water and centrifuged; the upper, colored organic phase was collected. This washing and collection was repeated until the extraction was complete. The pooled organic solvent evaporated under vacuum, and the residue was re-dissolved in 1 mL of hexane. The solution was filtered through a 0.45 µm membrane. All steps were conducted under subdued light to minimize photodegradation. The total carotenoid content was determined by measuring the absorbance of the hexane solution at 485 nm [19]. For the identification of individual carotenoids, the extract was analyzed by reversed-phase HPLC (Agilent 1100 series) with a C18 column (C18, 5 µm, 250 × 4.6 mm, Diamonsil plus, Cat# 99403) (Agilent Technologies, Santa Clara, CA, USA). The mobile phase consisted of (A) acetonitrile: water (9:1, *v*/*v*) and (B) ethyl acetate containing 1% formic acid. The flow rate was 0.5 mL/min, and detection was performed at 501 nm.

The column temperature was maintained at 25 °C, and 40 µL of sample was injected. The separation used a gradient program: 0–5 min, 100% A; 5–15 min, a linear increase to 100% B; 15–20 min, a return to 100% A, based on a method from the literature [20]. Peaks were identified by comparing retention times to commercial standards (β-carotene and γ-carotene (Sigma-Aldrich, St. Louis, MO, USA)) and to purified in-house standards (torulene and torularhodin) [20].

### 2.5. Transcriptomic Sequencing

To capture the transcriptional basis of the physiological states described above, total RNA was extracted from each single-batch bioreactor cultivation (C, HLP, HCP) for sequencing. It is important to note the design rationale: generating tightly controlled, reproducible physiological states for meaningful transcriptomic comparison in bioreactors is resource intensive. Therefore, this work was conceived as a foundational, in-depth case study. A total of 10 mg of yeast cells underwent RNA extraction using TRIzol^®^ Reagent (Thermo Fisher Scientific Inc., Waltham, MA, USA), following the instructions provided by the manufacturer (Invitrogen, Waltham, MA, USA). Subsequently, DNase I was used to remove genomic DNA, which was fragmented throughout the process. Subsequently, the RNA quality was assessed utilizing a 2100 Bioanalyzer (Agilent Technologies, Santa Clara, CA, USA), and its quantity was measured using the ND-2000 (NanoDrop Technologies, Wilmington, DE, USA). An RNA sample of high quality is utilized for the construction of a sequencing library. The RNA-seq transcriptome libraries were generated using the TruSeqTM RNA sample preparation kit from Illumina (San Diego, CA, USA), using 1 μg of total RNA as input. In brief, messenger RNA was extracted using polyA selection via oligo (dT) beads and subsequently fragmented using a fragmentation buffer. The process involved in this experiment included cDNA synthesis, end repair, A-base addition, and ligation of the Illumina-indexed adaptors, following the methodology provided by Illumina. Libraries were chosen to contain cDNA target fragments with a size range of 200–300 bp. These fragments were amplified using Phusion DNA Polymerase (NEB) via 15 PCR cycles. The TBS380 quantified the samples, and then Shanghai Biozeron Biotechnology Co., Ltd. (Shanghai, China) sequenced the paired-end libraries using the Illumina HiSeq PE 2 × 150 bp read length. The unprocessed paired-end readings underwent trimming and quality checking using Trimmomatic (V0.39) with the default parameters [21]. Subsequently, the process of filtering raw data (raw reads) involved the elimination of reads that contained adapters, poly-N, and sequences with subpar quality, resulting in clean data (clean reads). Ultimately, the RNA de novo assembly using Trinity (v2.15.0) [21] was performed using the clean data obtained from all samples. Gene function was determined by annotating it using various databases such as NCBI protein nonredundant (NR), String, KOG/COG (clusters of orthologous groups of proteins), the GO (gene ontology), and the Kyoto Encyclopedia of Genes and Genomes (KEGG). This annotation was performed by comparing the given transcripts with known proteins using BLASTX (V 2.16.0+) to identify the proteins with the highest sequence similarity [22]. A cut-off E-value of less than 1.0 × 10^−5^ was used to retrieve the function annotations. The estimation of gene expression levels was performed using the RNA-seq by the Expectation-Maximization (RSEM) method [23] for each sample. Genes that exhibited a fold change of 1 or higher and FDR ≤ 0.05 were identified as differentially expressed genes (DEGs). The differential expression analysis was performed using the EdgeR software package, which stands for Empirical Analysis of Digital Gene Expression in R (V2.12) (http://www.bioconductor.org/packages/2.12/bioc/html/edgeR.html, accessed on 21 December 2025). The RNA-seq data, consisting of the unprocessed reads and processed reads from the three samples, was deposited in the NCBI Sequence Read Archive (SRA) database. The corresponding accession numbers can be found in Table 2.

### 2.6. Statistical Analysis

Experiments were performed in triplicate, and data is expressed as the mean ± standard deviation.

## 3. Result and Discussion

### 3.1. Biomass, Lipid, and Carotenoid Production by R. glutinis Under Different Fed-Batch Fermentation Strategies

The manipulation of culture medium composition successfully induced distinct metabolic shifts in *R. glutinis*, leading to three unique physiological states: high biomass (control), high lipid (HLP), and high carotenoid (HCP) production. This targeted approach demonstrates the potential for steering microbial metabolism towards desired bioproducts.

**Figure 1:**
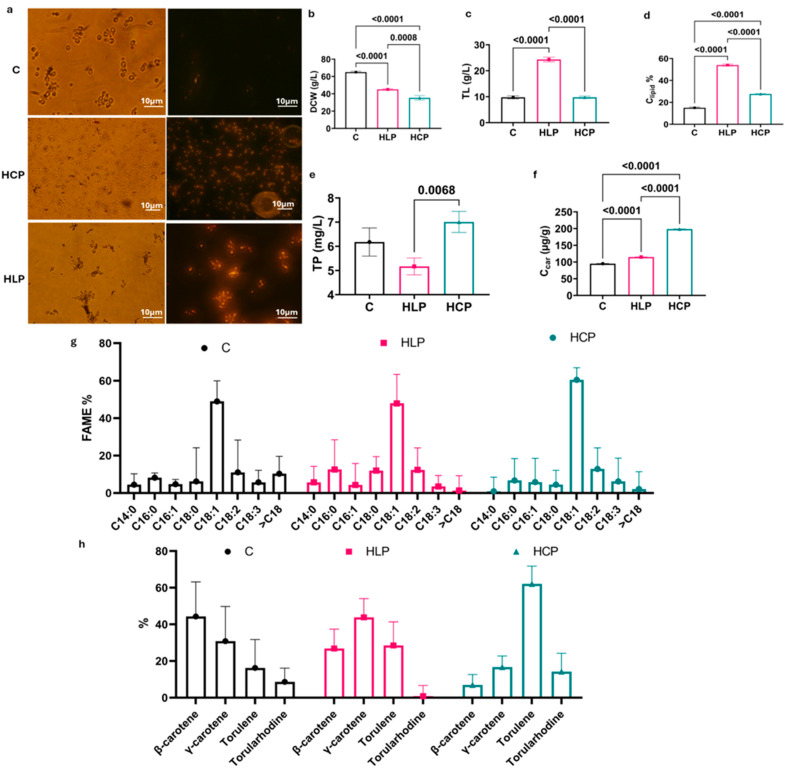
Detection of different physiological parameters of the control (C) group, high lipids production (HLP) group, and high carotenoid production group (HCP); (**a**) microscopic examination of the Nile red-stained cells after 96 h cultivation. The left column is the light illumination image, and the right column is the blue light illumination image. The scale bar is 10 µm. Effect of different medium compositions on (**b**) dry cell weight (DCW), (**c**) total lipids (TL), (**d**) cellular lipids (Clipid), (**e**) total pigment (TP), (**f**) cellular carotenoids (CCar), and (**g**) fatty acid methyl ester profile using GC for identification. (**h**) Individual carotenoids ratio using HPLC for identification. The results represent the mean values of three independent biological replicates, with variability expressed as standard deviation and displayed as error bars on the figures. Statistical significance was determined using one-way analysis of variance (ANOVA), with specific differences between group means identified using Tukey’s post hoc test for multiple comparisons.

Microscopic analysis of Nile red-stained cells provided a direct visual confirmation of these metabolic states (Figure 1a). All cells exhibited a typical oval morphology. Also, lipid body abundance varied significantly between conditions. Crucially, the HLP group triggered the most pronounced accumulation of intracellular lipid bodies, as indicated by the prominent yellow fluorescence. This visual evidence aligns quantitatively with the lipid yield of 24 ± 0.9 g/L (54% C_lipid_) from the HLP group, which represents a 3.6-fold (*p* < 0.0001) and 2-fold (*p* < 0.0001) increase over the control and HCP groups, respectively (Figure 1c,d). This massive lipid accumulation is a classic oleaginous response to multiple nutrient limitations (N, S, P), effectively creating a high carbon-to-nitrogen ratio that redirects metabolic flux from growth towards lipogenesis [11,13,24,25,26].

Conversely, biomass production (DCW) was primarily governed by nitrogen availability, a fundamental driver of microbial growth. The control group, with the highest nitrogen concentration, reached 65 ± 1.0 g/L. This yield decreased 1.4-fold (*p* < 0.0001) in the HLP group and 1.8-fold (*p* < 0.0001) in the HCP group (Figure 1b), consistent with their nutrient-limited conditions. This underlines a key trade-off in microbial bioprocessing: maximizing biomass often comes at the expense of secondary metabolite production. The paramount importance of nitrogen feeding is exemplified by Dias et al. [27], who achieved a biomass concentration of 127 g/L in *Rhodosporidium toruloides* through sophisticated fed-batch nitrogen supplementation.

While nitrogen limitation with certain metal supplementation in HLP favored lipids [2], the specific stress of aluminum sulfate and magnesium sulfate supplementation in the HCP group selectively enhanced carotenoid synthesis [2,5]. The HCP group achieved the highest total carotenoid titer at 7 ± 0.4 mg/L and a cellular content of 198.5 µg/g_DCW_ (Figure 1e,f), representing a significant 2.1-fold (*p* < 0.0001) and 1.7-fold (*p* < 0.0001) increase over the control and HLP groups, respectively. Beyond the total yield, a more striking finding was the nutrient-dependent reprogramming of the carotenoid pathway itself (Figure 1h).

The dominant carotenoid shifted from β-carotene (44.3%) in the nitrogen-sufficient control to γ-carotene (43.83%) under the multi-nutrient limitation of HLP, and finally to torulene (62.13%) under the specific ionic stress of HCP. This suggests that different stress signals precisely modulate the activity of enzymes like phytoene desaturase or lycopene cyclase, redirecting metabolic flux down specific branches of the carotenoid’s pathway [11,28,29].

This plasticity in carotenoid composition is a well-known adaptive response. Our results in *R. glutinis* align with findings in *Sporidiobolus pararoseus*, where Han et al. [30] reported a shift from β-carotene to torulene under nitrogen deficiency. Furthermore, the specific ion effect we observed is supported by Bhosale and Gadre [29] and Elfeky et al. [2,11], who demonstrated that divalent cations could alter carotenoid ratios, confirming that ionic environment is a critical lever for metabolic engineering.

The fatty acid profile was also significantly influenced by the cultivation strategy. Oleic acid (C18:1) was the most prevalent fatty acid in all samples, consistent with the literature on *R. glutinis* [6,8]. However, its proportion was highest in the HCP sample (60.5%) compared to the control (49%). More notably, the overall saturation balance was severely affected. The HLP regimen produced a more saturated profile (30% SFA). In contrast, the HCP treatment yielded a lipid profile highly enriched in unsaturated fatty acids (~85% UFA), compared to ~70.6% in the control (Figure 1g). This suggests that the sulfate/aluminum stressor not only triggers carotenogenesis but also profoundly impacts the redox state and desaturation pathways within the cell, prioritizing the production of unsaturated lipids.

### 3.2. Differential Gene Expression (DEG)

**Figure 2:**
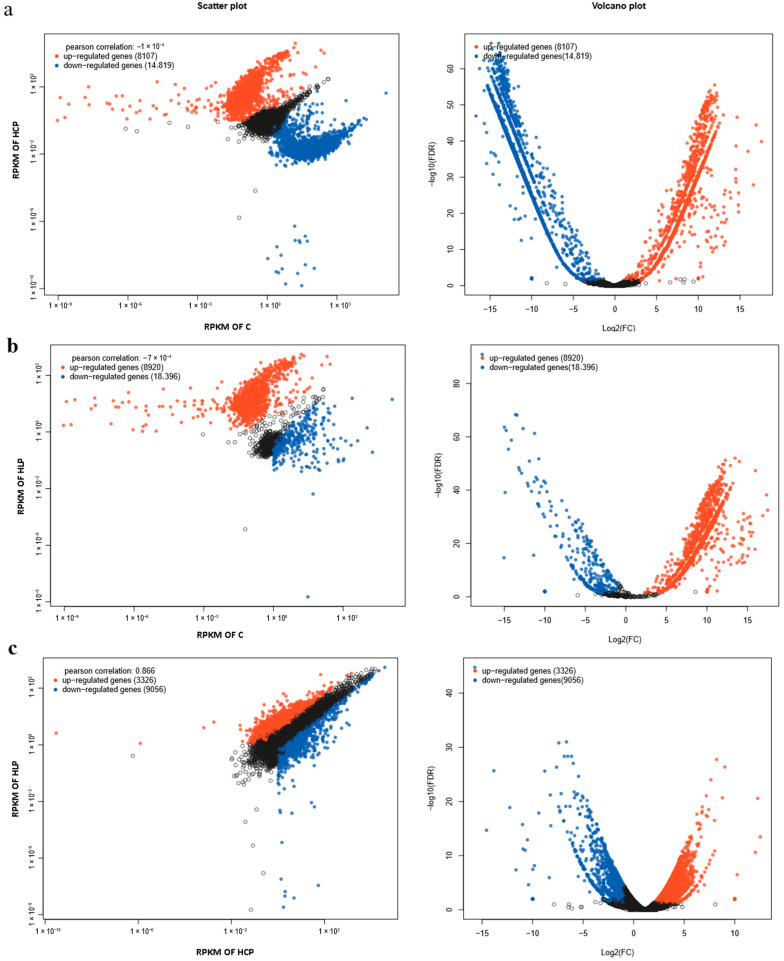
The expression of differential gene information statistics (FDR ≤ 0.05 and |log_2_(FC)| ≥ 1) (**a**–**c**).

RNA-seq analysis revealed extensive transcriptional reprogramming in *R. glutinis* in response to the different nutrient stresses, providing a molecular rationale for the observed metabolic shifts (Figure 2). The scale of differential gene expression (DEG) underscores the distinct physiological states induced by each condition.

The most dramatic transcriptional change occurred in the high lipid (HLP) condition. When compared to the nitrogen-replete control (C), a substantial 18,396 genes were downregulated alongside 8920 upregulated genes. This widespread downregulation is consistent with a global cellular response to multi-nutrient (N, S, P) limitation, where energy-intensive processes like growth and proliferation are suppressed. The concurrent upregulation of several thousand genes likely harbors key regulators of lipogenesis, channeling carbon flux towards triacylglycerol accumulation.

A similarly significant, though less extensive, shift was observed in the high carotenoid (HCP) group versus the control, with 14,819 genes downregulated and 8107 upregulated. This indicates that aluminum sulfate stress also triggers a major transcriptional response, but one that is distinct from multi-nutrient limitation. The specific set of genes altered in this comparison is presumably enriched for those involved in stress response and the carotenoid biosynthetic pathway.

Most instructively, a direct comparison between the HLP and HCP groups yielded a smaller, yet significant, set of 12,382 DEGs, with a strong bias towards downregulation in HLP (9056 down vs. 3326 up in HLP). This pattern suggests that the lipid-production regime enacts a more pronounced suppression of a broader range of cellular functions compared to the carotenoid production regime. This extensive transcriptional silencing in HLP may be a prerequisite for the massive reallocation of resources required for lipid overproduction. This finding aligns with the established paradigm of nutrient stress-induced lipid accumulation in oleaginous yeasts, often described as a “metabolic switch.” The data strongly supports the model proposed by Ratledge [31], who emphasized that nitrogen starvation halts cell proliferation, leading to the cessation of nucleotide and protein synthesis. This, in turn, redirects the cellular carbon flux, primarily from the citric acid cycle towards the overproduction of citrate, which is then cleaved in the cytosol to provide acetyl-CoA for de novo fatty acid synthesis. The massive downregulation observed here (18,396 genes) is the transcriptional manifestation of this halted proliferation and repression of non-essential anabolic processes. The fact that the HLP condition (multi-nutrient stress) shows a more severe global repression than HCP (aluminum sulfate stress) is logical; multi-nutrient limitation represents a more fundamental threat to cellular integrity, forcing a more conservation of energy and comprehensive metabolic reorientation to store carbon in its most energy-dense form, lipids.

### 3.3. Gene Ontology (GO) Enrichment Analysis

**Figure 3:**
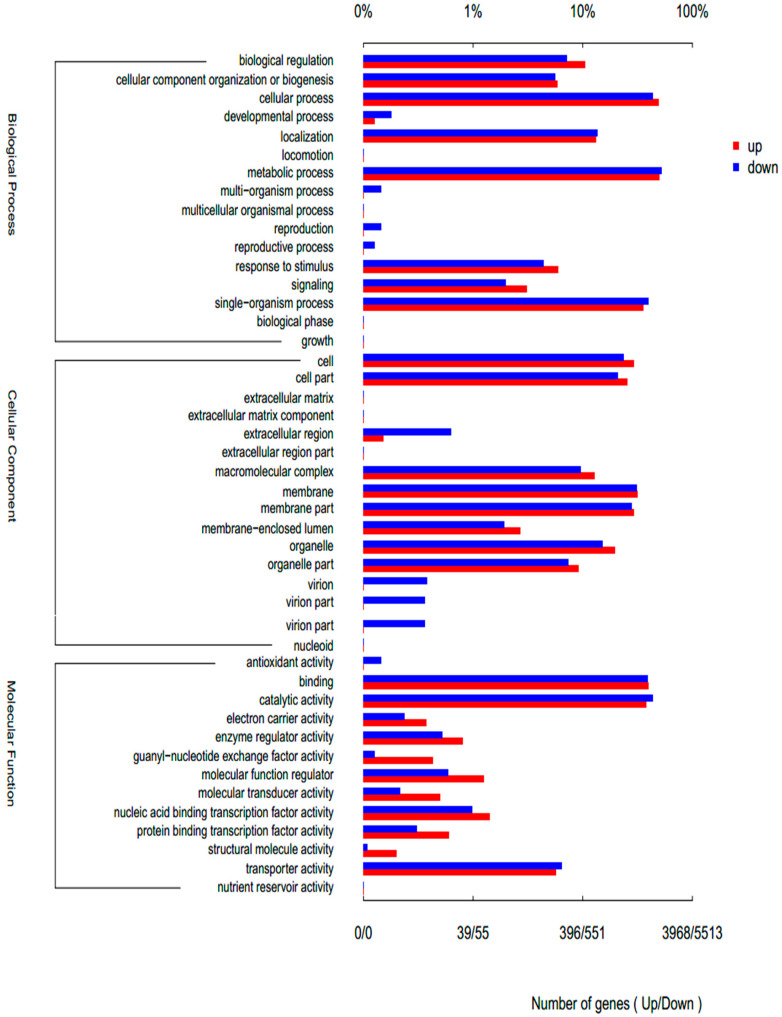
Number of up- and downregulated gene differences with HLP versus C, depending on GO annotation term. The lower abscissa in the figure indicates the number of genes annotated to a GO term, and the upper abscissa indicates the ratio of the number of genes annotated to a GO term to the total number of all GO annotated genes. Gene and GO term is a many-to-many relationship; that is, a gene can contain annotations for multiple GO terms, and a certain GO term will also correspond to multiple genes, not a one-to-one relationship.

**Figure 4:**
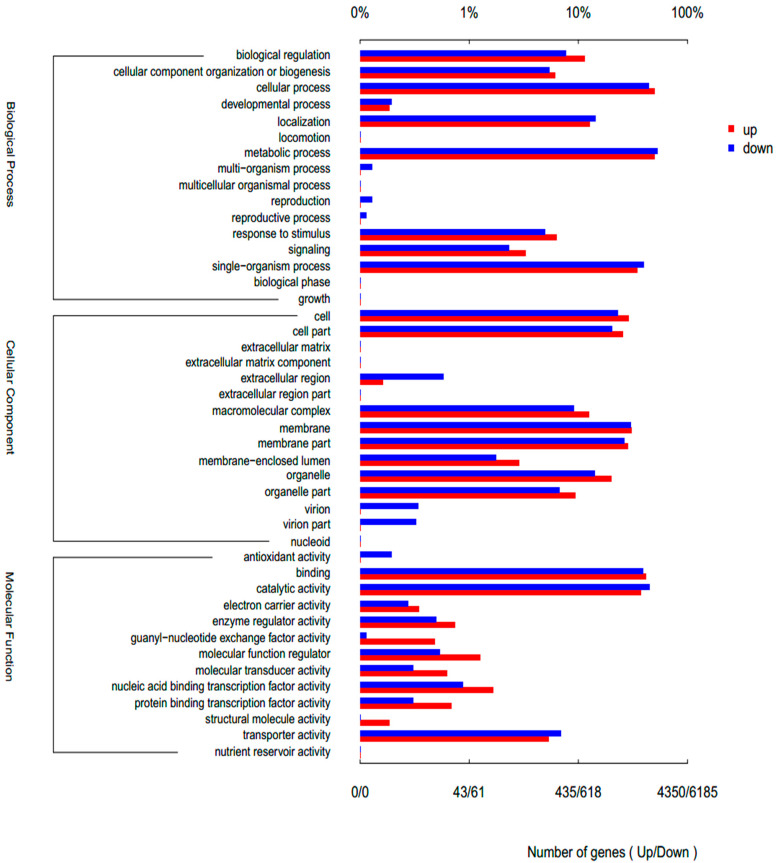
Number of up- and downregulated gene differences with HCP versus C, depending on GO annotation term.

**Figure 5:**
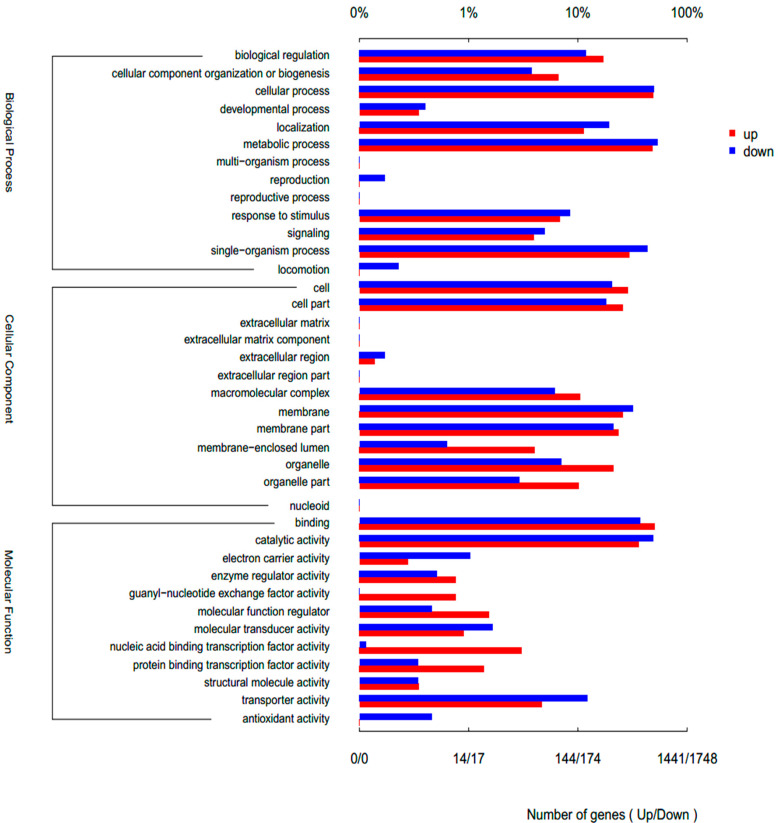
Number of up- and downregulated gene differences with HLP versus HCP, depending on GO annotation term. The lower abscissa in the figure indicates the number of genes annotated to a GO term, and the upper abscissa indicates the ratio of the number of genes annotated to a GO term to the total number of all GO annotated genes. Gene and GO term is a many-to-many relationship; that is, a gene can contain annotations for multiple GO terms, and a certain GO term will also correspond to multiple genes, not a one-to-one relationship.

Gene Ontology (GO) enrichment analysis of the differentially expressed genes provided a functional context for the metabolic shifts observed, revealing how distinct nutrient stresses rewire cellular processes at a systemic level (Figure 3, Figure 4 and Figure 5). The transcriptomic profiles of *Rhodotorula glutinis* under high lipid (HLP) and high carotenoid (HCP) production conditions, each compared to the control (C), reveal a foundational shift from growth to storage, executed with distinct transcriptional strategies (Figure 3 and Figure 4). Both HLP and HCP, relative to the control, showed significant upregulation of stress-responsive processes such as response to stimulus, signaling, and biological regulation, a well-documented response where nutrient limitation is perceived as a major stressor triggering defense mechanisms [32]. This reprogramming facilitates the activation of storage compound biosynthesis, with lipids and carotenoids serving as critical survival reserves under different stress conditions [28,33]. Relative to the control, HLP induces a profound, specialized lipid-factory state. This is driven by a massive downregulation of general metabolic process (GO:0008152) genes (3266 down) paired with the targeted upregulation of anabolic modules (2170 up). Growth is systematically suppressed, evidenced by net downregulation in single-organism process (2461 down vs. 1502 up), cellular process (2751 down vs. 2155 up), and developmental/reproductive categories. This global suppression of non-essential functions, a strategy to conserve energy and channel carbon flux singularly toward lipid biosynthesis, mirrors observations in other yeasts under nutrient limitation [34,35]. Cellular architecture is transformed, with a near-equal but oppositional shift in organelle genes (870 down vs. 871 up) favoring ER, mitochondria, and lipid droplets [36], and a net reduction in membrane genes (1879 down vs. 1325 up) indicating metabolic closure. Enzymatically, catalytic activity is streamlined (2755 down vs. 1628 up), downregulating diverse enzymes while upregulating lipid-specific enzymes, which are core to fatty acid and triacylglycerol assembly. This specialized state is underscored by a strong downregulation of antioxidant activity (12 down vs. 3 up) to redirect NADPH—a critical cofactor for lipid synthesis [37]—and a marked upregulation of guanyl-nucleotide exchange factor activity (21 up vs. 7 down) to facilitate lipid droplet trafficking, a process essential for lipid droplet biogenesis [38] (Figure 3).

The HCP condition also initiates a storage shift but with a different product focus. It shows substantial downregulation in metabolic process (2890 down), with concurrent upregulation (1999 up) for biosynthesis. Similar downregulation of growth-focused single-organism process occurs. Cellular restructuring involves a net downregulation of organelle genes (842 down vs. 781 up) and membrane genes (1710 down vs. 1258 up). Its enzymatic profile in catalytic activity (2412 down vs. 1503 up) is tailored for the isoprenoid pathway, upregulating transferases crucial for lipid and carotenoid synthesis. Unlike HLP, HCP reconfigures rather than suppresses its response to stimulus (243 down vs. 236 up), likely adapting to metal stress and utilizing carotenoid production as a core antioxidant defense [28,33] (Figure 4).

Based on the direct transcriptomic comparison between HLP and HCP, the distinct metabolic specializations for lipid versus carotenoid production are clear (Figure 5). While both conditions repress growth-related processes, HLP demonstrates a more focused and aggressive reprogramming toward becoming a dedicated lipid factory. This is evident in its stronger overall suppression of general cellular processes and cellular component organization, alongside a profound reconfiguration of core functions. Key cellular architecture is strategically reshaped: membrane-related genes are restructured (373 up, 556 down), likely to favor the endoplasmic reticulum and mitochondria for lipid synthesis [36]; organelles are strongly upregulated (305 up, 124 down); and membrane-enclosed lumens are enhanced (58 up, 11 down) to support compartmentalization. At the molecular level, catalytic activity is streamlined (518 up, 860 down) to prioritize linear biosynthetic enzymes, while transporter activity is dramatically reduced (67 up, 215 down), reflecting metabolic closure commonly seen in storage-phase microbes [39]. Comprehensive regulatory rewiring is also prominent, with strong induction of DNA-binding transcription factors (44 up, 2 down) and transcription co-regulators (20 up, 6 down), indicative of a major transcriptional overhaul. Condition-specific signatures further define HLP’s specialized state, including a pronounced upregulation of guanyl-nucleotide exchange factor activity (11 genes up in HLP vs. 1 in HCP) for vesicular trafficking [36], upregulation of molecular function regulators (22 up, 8 down), and a stark downregulation of antioxidant activity (8 down) to redirect NADPH toward lipid synthesis, a trade-off observed in other high lipid-producing organisms [34,35]. Together, these changes illustrate HLP’s coordinated shift to a high-efficiency lipid-factory state, activating anabolic pathways and specialized compartments while suppressing growth, stress responses, and competing metabolism to maximize carbon flux into storage lipids.

Furthermore, the reprogramming of electron carrier activity reveals a condition-specific strategy for redirecting redox metabolism. Compared to the control, HLP shows net upregulation (17 genes up vs. 15 down) to supply NADPH for lipid biosynthesis (Figure 3). Similarly, HCP exhibits a balanced reorganization (15 genes up vs. 13 down) to support both lipid and carotenoid synthesis (Figure 4). Crucially, electron carrier activity was significantly more upregulated in HCP than in HLP, where HLP demonstrates a markedly streamlined redox network (Figure 5). This provides a direct transcriptional explanation for the observed metabolic output: carotenoid biosynthesis (HCP) is more redox-intensive than lipid biosynthesis (HLP), requiring a greater flux of NADPH to synthesize its products, a known constraint in microbial systems [40,41] (Figure 5).

The lower abscissa in the figure indicates the number of genes annotated to a GO term, and the upper abscissa indicates the ratio of the number of genes annotated to a GO term to the total number of all GO annotated genes. Gene and GO term is s many-to-many relationship; that is, a gene can contain annotations for multiple GO terms, and a certain GO term will also correspond to multiple genes, not a one-to-one relationship.

### 3.4. Central Carbon Metabolic Pathways

Transcriptomic analysis revealed condition-specific reprogramming of central carbon metabolism in *R. glutinis* (Table 3), which delineates the central carbon metabolic pathways driving the divergence towards either lipid biosynthesis (HLP), carotenogenesis (HCP), or proliferative growth (C).

Glycolytic flux was a major point of divergence. A key difference was the strong, significant upregulation of the NADP+-dependent glyceraldehyde-3-phosphate dehydrogenase (gapN) in the HCP condition (Log_2_FC(HCP/C) = 12.24, Log_2_FC (HCP/HLP) = 11.8, *p* = 0.000), providing a mechanism for direct NADPH generation to meet the high redox demand of carotenogenesis. Its expression showed nonsignificant change in HLP/C. Conversely, the significant upregulation of phosphoenolpyruvate carboxykinase (PEPCKA, Log_2_FC(HLP/HCP) = 10.90, *p* = 0.000) and pyruvate kinase (PK, Log_2_FC(HLP/HCP) = 10.70, *p* = 0.004) in HLP indicates a reinforced push to generate pyruvate, the primary precursor for acetyl-CoA [40].

The conversion of pyruvate to cytosolic acetyl-CoA was a key step. The upregulation of the pyruvate dehydrogenase complex (PDC, Log_2_FC(HLP/HCP) = 3.000, *p* = 0.000) suggests enhanced flux through this reaction to generate acetyl-CoA, the essential precursor for lipogenesis. Furthermore, in oleaginous yeasts, NADH produced by the PDC provides crucial reducing equivalents that can be used to generate the NADPH required for fatty acid biosynthesis via transhydrogenase cycles [29]. While comparing high C/N ratio treatments with low C/N ratio, ATP-citrate lyase (ACL) was also significantly upregulated (Log_2_FC ~6.7–7.9, *p* = 0.000), as reported in similar studies [42,43,44].

Consistent with oleaginous metabolism, the TCA cycle was transcriptionally suppressed in both high C/N ratio production conditions compared to the control, evidenced by the significant downregulation of isocitrate dehydrogenase (IDH1, Log_2_FC(HLP/C) = −12.39, *p* = 0.000; Log_2_FC(HCP/C) = −12.62, *p* = 0.000) and citrate synthase (CS, Log_2_FC(HLP/HCP) = −11.02, *p* = 0.000), signaling a diversion of acetyl-CoA away from respiration and toward storage compound assembly [13,44].

The pentose phosphate pathway (PPP) was prioritized for NADPH generation in the HCP condition. Enzymes including glucose-6-phosphate 1-dehydrogenase (zwf), 6-phosphogluconate dehydrogenase (GND), and transketolase (tktA) were all significantly downregulated in HLP compared to HCP (Log_2_FC ~ −10.0 to −10.9, *p* ≤ 0.005), indicating that the PPP route is a crucial source of reducing power for carotenoid overproduction [43].

### 3.5. Lipid Metabolism

Transcriptional data provides a clear explanation for the high lipid yield in the HLP group, showing coordinated upregulation of biosynthesis and downregulation of degradation (Table 4).

The commitment step, catalyzed by acetyl-CoA carboxylase (ACC), was significantly upregulated in HCP versus control (Log_2_FC(HCP/C) = 10.06, *p* = 0.000) and the HCP group (Log_2_FC = 5.033, *p* = 0.000), confirming its role as a crucial regulatory enzyme [45]. This universal upregulation of ACC across stress conditions indicates it is a common gatekeeper for enhanced carbon flux into fatty acid synthesis, a well-documented response to nitrogen limitation in oleaginous species [45]. The most decisive finding was the specific upregulation of the fungal-type fatty acid synthase complex in HLP (FAS1 Log_2_FC(HLP/HCP) = 4.853, *p* = 0.000; FAS2 Log_2_FC(HLP/HCP) = 2.990, *p* = 0.000), confirming targeted activation of the primary machinery for converting malonyl-CoA into C16/C18 fatty acids under multi-nutrient limitation [13]. The stark contrast in FAS expression between HLP and HCP underscores that multi-nutrient stress provides a more potent and specific signal for de novo lipogenesis than aluminum sulfate stress. Concurrently, fatty acid degradation (β-oxidation) was strongly suppressed in HLP. Key enzymes acd (Log_2_FC(HLP/HCP) = −10.55); echA (Log_2_FC(HLP/HCP) = −10.51); and fadA, (Log_2_FC(HLP/HCP) = −9.703) were significantly downregulated, preventing the catabolism of newly synthesized fatty acids and channeling carbon toward storage lipid accumulation. This coordinated regulation—simultaneous induction of anabolism and repression of catabolism—represents a classic “futile cycle” avoidance strategy, ensuring maximum metabolic efficiency for converting carbon into storage lipids [46]. The data also reveals a shift in the site of fatty acid management. The data also reveals a shift in the site of fatty acid management. Enzymes associated with mitochondrial acyl metabolism (MECR, Log_2_FC(HLP/C) = 15.24; HADH, Log_2_FC(HLP/C) = 8.803) were upregulated in HLP, suggesting enhanced mitochondrial activity to support high-rate lipid synthesis. Meanwhile, enzymes for very-long-chain fatty acid (VLCFA) synthesis in the endoplasmic reticulum (KCS, Log_2_FC(HLP/HCP) = −11.64; ACOT, Log_2_FC(HLP/C) = −13.35) were downregulated. This suggests carbon is prioritized for storage lipid production over complex membrane lipid synthesis in HLP. This reprioritization is a critical adaptation; under nutrient stress, the cell halts growth, and the demand for new membrane phospholipids diminishes. By downregulating ER-based VLCFA synthesis, the cell efficiently shunts resources away from structural components and towards energy storage, a phenomenon consistent with the metabolic rewiring observed in other oleaginous fungi [47].

### 3.6. Carotenoid Biosynthetic Pathway

The transcriptional profile reveals a complex regulatory landscape explaining the distinct carotenoid profiles (Table 5). A critical bottleneck was observed at the mevalonate pathway entry. Acetyl-CoA C-acetyltransferase (atoB) was downregulated in HLP versus HCP (Log_2_FC = −10.62, *p* = 0.000), and HMG-CoA reductase (HMGCR) showed only modest upregulation in HCP versus HLP (Log_2_FC(HLP/HCP) = 2.284, *p* = 0.008), suggesting limited flux into mevalonate [48].

Downstream, the pathway exhibited a paradoxical pattern: phosphomevalonate kinase (mvaK2) was upregulated in the high C/N ratio groups versus control (Log_2_FC ~14.9, *p* = 0.000), but mevalonate kinase (mvaK1, Log_2_FC = −15.04) and diphosphomevalonate decarboxylase (MVD, Log_2_FC = −12.63) were downregulated, suggesting potent post-transcriptional regulation restricting the Isoprenoid pathway (IPP) pool [40]. Despite upstream constraints, a push toward carotenogenesis in HCP was evident. Farnesyl diphosphate synthase (FDPS) was downregulated in HLP versus HCP (Log_2_FC = −0.870, *p* = 0.000), indicating a relative advantage for granyl granyl perophsphate (GGPP) production in HCP [48]. The subtle but significant downregulation of FDPS in HLP may serve to conserve the limited IPP pool for sterol synthesis, while in HCP, more carbon can be allocated to the carotenoid branch point.

The most striking finding was in the carotenogenic pathway itself. The enzymes for β-carotene production, phytoene synthase (crtB) and desaturase (AL1) [49], were downregulated in HLP and HCP versus control (Log_2_FC ~ −12.4 to −14.2, *p* = 0.000). Conversely, beta-carotene hydroxylase (crtZ) was significantly downregulated in HLP versus HCP and C (Log_2_FC~ −10.65 and −10.80, respectively, *p* = 0.000), torulene-rich condition. This finding supports a novel role for CrtZ in *R. glutinis* in the hydroxylation of torulene to torularhodin, aligning with findings in engineered *E. coli* [50,51]. This proposed function for CrtZ expands its canonical role beyond hydroxylating β-carotene to torulene and suggests a metabolic shortcut for torularhodin synthesis that bypasses γ-carotene, a finding that aligns with recent hypotheses about carotenoid diversity in red yeasts [52].

Finally, an inverse relationship between the regulatory enzymes of fatty acid synthesis and those of the isoprenoid pathway was observed, highlighting a metabolic trade-off that governs carbon partitioning in *R. glutinis* towards either lipids or carotenoids. This competitive partitioning of acetyl-CoA, the central precursor for both pathways, creates a natural tug-of-war, and our transcriptomic data provides a molecular basis for this well-documented physiological phenomenon in oleaginous yeasts. The data suggests aluminum sulfate stress (HCP) partially alleviates the severe repression seen in multi-nutrient stress (HLP), allowing for a moderate flux into the carotenoid branch without triggering the full lipogenic program.

## 4. Conclusions

This study employed a multi-pronged fed-batch strategy to steer the metabolism of *Rhodotorula glutinis* towards distinct high-yield states for biomass, lipids, or carotenoids. Our integrated physiological and exploratory transcriptomic analysis provides evidence that these product divergences are associated with fundamentally different metabolic and regulatory programs triggered by specific nutrient stresses. It is important to emphasize that the transcriptomic findings are derived from a single biological instance per condition and thus represent a foundational case study for generating specific hypotheses.

Multi-nutrient limitation (N, S, P) in the HLP condition induced a classic oleaginous response, as suggested by widespread transcriptional changes. This was marked by a dominant downregulation of growth-related gene categories and a concerted shift in central carbon metabolism consistent with the channeling of acetyl-CoA towards lipogenesis. The observed coordinated upregulation of de novo fatty acid synthesis (e.g., FAS), alongside the suppression of β-oxidation genes, presents a candidate mechanism for the efficient triacylglyceride accumulation that resulted in a high lipid yield of 24 g/L. In contrast, aluminum sulfate stress in the HCP condition appears to have triggered a more targeted response. Its hallmark was a heightened demand for NADPH, potentially met through the specific upregulation of the pentose phosphate pathway and the NADP+-dependent gapN. This putative redox-driving force was coupled with a precise reprogramming of the carotenoid pathway. The significant relative decrease in beta-carotene hydroxylase (CrtZ) expression in HLP, and its retention in HCP, suggests a novel and testable hypothesis regarding the enzyme’s role, potentially facilitating the direct conversion of torulene to torularhodin and explaining the dramatic product shift. Crucially, our transcriptomic data offers a prospective molecular basis for the metabolic tug-of-war between lipids and carotenoids, indicating an inverse relationship between the expression of fatty acid synthesis and isoprenoid pathway genes, which highlights the competitive partitioning of acetyl-CoA. The ionic environment emerges as a promising lever for manipulating the end-product profile. In summary, this work provides a robust foundational dataset and prioritizes key genetic targets (e.g., crtZ, gapN, FAS) for the future metabolic engineering of *R. glutinis*. The distinct regulatory patterns propose a model in which implementing a multi-nutrient limitation program could favor lipids, while an ion-specific stress response could favor carotenoids. The primary contribution of this study is the generation of precise, experimentally testable hypotheses. Future work must focus on the functional validation of these candidate DEGs through genetic manipulation and confirmatory studies, with full biological replication to construct advanced, reliably engineered microbial cell factories.

## 5. Limitations

This study provides a foundational transcriptional map of *Rhodotorula glutinis* under industrially relevant production regimes; however, some methodological constraints should be acknowledged. First, the conclusions are drawn from the analysis of a single biological replicate per condition. Second, the transcriptomic data represents a single time point (96 h) corresponding to peak product accumulation. While this captures the definitive high-yield state, it does not elucidate the dynamic transcriptional shifts leading to it. Finally, this work reports transcriptional findings, and RT-qPCR validation of key differentially expressed genes was not performed within the scope of this foundational study.

## Figures and Tables

**Table 1:** Medium composition and feeding solution of the different experimental groups.

Group	Medium Composition (g/L)	Metal	Feeding Solution (g/100 mL Distilled Water)	Feeding Rate (mL/h)	Residual Glucose (g/L)
C	Glucose (20), peptone (10), yeast extract (10)	None	Glucose: Yeast Extract (50:50)	5	>5.00
HLP	Glucose (60), yeast extract (5), (NH_4_)_2_SO_4_ (1), KH_2_PO_4_ (1.5), MgSO_4_ (1)	BaCl_2_ and MnCl_2_	Glucose (100)	4	>25.0
HCP	Glucose (60), yeast extract (5), (NH_4_)_2_SO_4_ (2), KH_2_PO_4_ (2), MgSO_4_ (3)	Al_2_SO_4_	Glucose: MgSO_4_ (100:0.20)	4	>25.0

**Table 2:** NCBI Sequence Read Archive (SRA) accession numbers of experimental groups.

Accession	Bioproject Accession	Biosample Accession	Group
SRR10738942	PRJNA596366	SAMN13620008	C
SRR10738940	PRJNA596366	SAMN13620010	HCP
SRR10738939	PRJNA596366	SAMN13620011	HLP

**Table 3:** Enzymes of glycolysis, TCA, and PPP full description and their statistical analysis.

	Symbol	Definition	HCP/C	HLP/C	HLP/HCP	Log2 FC HCP/C (*p* Value)	Log2 FC HLP/C (*p* Value)	Log2 FC HLP/HCP (*p* Value)
Glycolysis enzymes	PGM	phosphoglucomutase	U/D	U/D	D	-	-	−5.789 (0.001)
	GPI	glucose-6-phosphate isomerase	U	U/D	D	0.949 (0.005)	-	−10.46 (0.000)
	galM	aldose-1-epimerase	U/D	U	U	-	16.90 (0.000)	4.231 (0.000)
	ald	fructose biphosphate aldolase	U/D	U/D	D	-	-	−9.600 (0.014)
	gapA	glyceraldhyde-3-phosphate dehydrogenase	U/D	U/D	D	-	-	−10.00 (0.008)
	gapN	glyceraldhyde-3-phosphate dehydrogenase NADP+	U	N	D	12.24 (0.000)	-	−11.80 (0.000)
	PGK	phosphoglycerate kinase	U/D	U/D	D	-	-	−4.200 (0.000)
	gpmI	2,3 biphosphoglycerate-independent phosphoglycerate mutase	U/D	U/D	D	-	-	−10.10 (0.005)
	gpmA	2,3biphosphoglycerate-dependent phosphoglycerate mutase	U	U	N	7.700 (0.001)	8.800 (0.020)	-
	ENO	enolase	U/D	U/D	D	-	-	−8.000 (0.000)
	PEPCKA	phosphoenol pyruvate carboxykinase ATP	U/D	U/D	U	-	-	10.90 (0.000)
	PEPCKG	phosphoenol pyruvate carboxykinase GTP	U/D	D	D	-	−10.20 (0.005)	−9.900 (0.000)
	PK	pyruvate kinase	U/D	U/D	U	-	-	10.70 (0.004)
	PDC	pyruvate decarboxylase	U/D	U/D	U	-	-	3.000 (0.000)
	E1.2.1.5	aldehyde dehydrogenase (NAD(P)+)	D	D	N	−16.30 (0.000)	−16.30 (0.000)	-
	exaA	alcohol dehydrogenase (cytochrome c)	U/D	U	U	-	9.900 (0.005)	10.70 (0.000)
	ME-NAD	NAD-dependent malic enzyme, mitochondrial	U/D	U/D	U	-	-	2.200 (0.020)
	ME-NADP	malic enzyme-NADP dependent	U	U	D	10.56 (0.001)	11.95 (0.000)	−9.078 (0.014)
	MDH1	malate dehydrogenase mitochondrial	D	D	U	−9.969 (0.000)	−16.98 (0.000)	2.039 (0.035)
	MDH2	malate dehydrogenase, NAD-dependent (cytoplasmic)	U/D	U/D	U	-	-	2.541 (0.002)
	ACL	beta subunit citrate lyase	U	U	N	6.705 (0.000)	7.944 (0.000)	-
TCA cycle	CS	citrate synthase	U/D	U/D	D	-	-	−11.02 (0.000)
	IDH	Isocitrate dehydrogenase	D	D	N	−12.62 (0.000)	−12.39 (0.000)	-
	sucD	Succinyl-CoA synthetase alpha subunit	U	N	D	10.71 (0.014)	-	−1.898 (0.004)
	frdA	fumarate reductase flavoprotein subunit	N	D	D	-	−9.681 (0.014)	−9.590 (0.000)
PPP	zwf/G6PD	glucose-6-phosphate1-dehydrogenase	U/D	U/D	D	-	-	−10.87 (0.005)
	GND	6-phosphogluconate dehydrogenase	U/D	U/D	D	-	-	−10.06 (0.000)
	rpiA	ribose5-phosphate isomerase	U	N	D	11.39 (0.000)	-	−10.18 (0.005)
	rbsK	ribokinase	U/D	U/D	U	-	-	2.364 (0.000)
	RPE	ribulose-phosphate-3-epimerase	N	N	D	-	-	−9.230 (0.008)
	tktA	transketolase	N	N	D	-	-	−10.68 (0.004)

U/D means that some Blast hits showed upregulation and others showed downregulation. U means a significant upregulation, D means a significant downregulation, N means a non-significant reading, the *p*-value is more than 0.05, and (-) means Log_2_ FC was neglected as the difference between two samples is not statistically significant.

**Table 4:** Enzymes of fatty acid synthesis, fatty acid elongation, and fatty acid degradation full description and their statistical analysis.

Pathway	Symbol	Definition	HCP/C	HLP/C	HLP/HCP	Log_2_FC HCP/C (*p* Value)	Log_2_FC HLP/C (*p* Value)	Log_2_FC HLP/HCP (*p* Value)
Fatty acid synthesis	ACC	Acetyl CoA carboxylase	U	U/D	U	10.06 (0.000)	-	5.033 (0.000)
	FAS1	Fatty acid synthase subunit beta, fungi type	U/D	U/D	U	-	-	4.853 (0.000)
	FAS2	Fatty acid synthase subunit alpha, fungi type	U	U	U	11.16 (0.000)	18.18 (0.000)	2.990 (0.000)
Fatty acid elongation	ACAC2	acetyl-CoA acyltransferase 2	U	U	N	7.019 (0.000)	8.874 (0.000)	-
	HADH	3-hydroxyacyl-CoA dehydrogenase	N	U	N	7.872 (0.000)	8.803 (0.000)	-
	MECR	mitochondrial enoyl-[acyl-carrier protein] reductase/trans-2-enoyl-CoA reductase	U	U	N	14.20 (0.000)	15.24 (0.000)	-
	PPT	palmitoyl-protein thioesterase	N	N	U	-	-	2.782 (0.001)
	ACOT1_2_4	acyl-coenzyme A thioesterase 1/2/4	D	D	N	−13.35 (0.000)	−13.35 (0.000)	-
	KCS	3-ketoacyl-CoA synthase	N	D	D	-	−12.24 (0.004)	−11.64 (0.000)
	PHS1	very-long-chain (3R)-3-hydroxyacyl-CoA dehydratase	D	D	N	−12.91 (0.000)	−12.91 (0.000)	-
	ACSL, fadD	long-chain acyl-CoA synthetase	N	D	D	-	−10.19 (0.000)	−9.288 (0.002)
Fatty acid degradation	ACOX1	acyl-CoA oxidase	U/D	U/D	U	-	-	2.043 (0.038)
	acd	acyl-CoA dehydrogenase	N	D	D	-	−12.11 (0.008)	−10.55 (0.024)
	echA	enoyl-CoA hydratase	N	D	D	-	−11.53 (0.008)	−10.51 (0.004)
	HADH	3-hydroxyacyl-CoA dehydrogenase	U	U	N	7.872 (0.000)	8.803 (0.000)	-
	fadA	acetyl-CoA acyltransferase	N	N	D	-	-	−9.703 (0.043)
	atoB	acetyl-CoA C-acetyltransferase	N	N	D	-	-	−10.62 (0.000)
Glycerophospholipid pathway	GPD1	glycerol-3-phosphate dehydrogenase (NAD+)	N	N	D	-	-	−8.774 (0.008)
	glpA	glycerol-3-phosphate dehydrogenase	N	N	D	-	-	−9.185 (0.004)
	pgsA	CDP-diacylglycerol---glycerol-3-phosphate 3-phosphatidyltransferase	D	D	N	−15.34 (0.000)	−15.34 (0.000)	-
	GEP4	phosphatidylglycerophosphatase GEP4	N	N	D	-	-	−2.357 (0.007)
	plc	phospholipase C	D	D	U	−12.39 (0.000)	−12.39 (0.000)	2.414 (0.006)
	GDE1	glycerophosphodiester phosphodiesterase	N	N	U	-	-	2.357 (0.001)
	CKT1	choline kinase	N	N	U	-	-	3.400 (0.000)
	clsA_B	cardiolipin synthase A/B	N	N	D	-	-	−10.02 (0.001)
	PCYT1	choline-phosphate cytidylyltransferase	D	D	N	−11.94 (0.000)	−11.94 (0.000)	-

U/D means some Blast hits showed upregulation and others showed downregulation. U means a significant upregulation, D means a significant downregulation, N means a non-significant reading, the *p*-value is more than 0.05, (-) means Log_2_ FC was neglected as the difference between two samples is not statistically significant.

**Table 5:** Enzymes of carotenoids pathway full description and their statistical analysis.

Pathway	Symbol	Definition	HCP/C	HLP/C	HLP/HCP	Log_2_FC HCP/C (*p* Value)	Log_2_FC HLP/C (*p* Value)	Log_2_FC HLP/HCP (*p* Value)
Mevalonate	atoB	acetyl-CoA C-acetyltransferase	N	N	D	-	-	−10.62 (0.000)
	HMGCR	hydroxymethylglutaryl-CoA reductase (NADPH)	U/D	U/D	U	-	-	2.284 (0.008)
	MVK, mvaK1	mevalonate kinase	D	D	N	−15.04 (0.000)	−15.04 (0.000)	-
	mvaK2	phosphomevalonate kinase	U	U	U	14.89 (0.000)	14.92 (0.000)	0.026 (0.000)
	MVD, mvaD	diphosphomevalonate decarboxylase	D	D	N	−12.63 (0.000)	−12.63 (0.000)	-
Isoprenoid	IDI	isopentenyl-diphosphate delta-isomerase	U/D	D	D	-	−8.907 (0.025)	−9.527 (0.038)
	FDPS	farnesyl diphosphate synthase	U/D	U/D	D	-	-	−0.87
	hexPS, COQ1	prenyl cysteine oxidase/farnesylcysteine lyase	U/D	U/D	U	-	-	2.219 (0.018)
Carotenoids	crtB	15-cis-phytoene synthase	D	D	N	−12.40 (0.000)	−12.40 (0.000)	-
	AL1	phytoene desaturase (3,4-didehydrolycopene-forming)	D	D	N	−14.16 (0.000)	−14.16 (0.000)	-
	AL2	15-cis-phytoene synthase/lycopene beta-cyclase	U/D	U/D	U	-	-	0.125 (0.047)
	crtZ	beta-carotene 3-hydroxylase	N	D	D	-	−10.80 (0.043)	−10.65 (0.001)

U/D means that some Blast hits showed upregulation, and others showed downregulation. U means a significant upregulation, D means a significant downregulation, N means a non-significant reading, p-value is more than 0.05, and (-) means Log2 FC was neglected as the difference between two samples is not statistically significant.

## Data Availability

All the data and materials used for the preparation of the manuscript are presented in it.

## Abbreviations

The following abbreviations are used in this manuscript:

*R. glutinis*	*Rhodotorula glutinis*
C/N	Carbon-to-nitrogen ratios
C	Control
HLP	High lipid production
HCP	High carotenoid production
FAS1/2	Fatty acid synthase complex
PPP	Pentose phosphate pathway
CrtZ	Putative beta-carotene hydroxylase
gapN	NADP+-dependent glyceraldehyde-3-phosphate dehydrogenase
C/S	Carbon to sulfur
DCW	Dry Cell Weight
TL	Total lipids
GC	Gas Chromatography
RSEM	Expectation-Maximization
DEG	Differentially expressed genes
SRA	Sequence Read Archive
C_lipid_	Cellular lipid
Ccar	Cellular Carotenoid
TP	Total Pigment

## References

[1] Elfeky N., Rizk A., Gharieb M.M. (2024). Exploring the lipids, carotenoids, and vitamins content of *Rhodotorula glutinis* with selenium supplementation under lipid accumulating and growth proliferation conditions. BMC Microbiol..

[2] Elfeky N., Elmahmoudy M., Bao Y. (2020). Manipulation of Culture Conditions: Tool for Correlating/Improving Lipid and Carotenoid Production by *Rhodotorula glutinis*. Processes.

[3] Kot A.M., Błażejak S., Kurcz A., Gientka I., Kieliszek M. (2016). *Rhodotorula glutinis*-potential source of lipids, carotenoids, and enzymes for use in industries. Appl. Microbiol. Biotechnol..

[4] Thancharoen K., Malasri A., Leamsingkorn W., Boonyalit P. (2017). Selection of Oleaginous Yeasts with Lipid Accumulation by the Measurement of Sudan Black B for Benefits of Biodiesel. Int. J. Pharm. Med. Biol. Sci..

[5] Zoz L., Carvalho J.C., Soccol V.T., Casagrande T.C., Cardoso L. (2015). Torularhodin and torulene: Bioproduction, properties and prospective applications in food and cosmetics—A review. Braz. Arch. Biol. Technol..

[6] Tkáčová J., Čaplová J., Klempová T., Čertík M. (2017). Correlation between lipid and carotenoid synthesis in torularhodin-producing *Rhodotorula glutinis*. Ann. Microbiol..

[7] Gong G., Liu L., Zhang X., Tan T. (2019). Comparative evaluation of different carbon sources supply on simultaneous production of lipid and carotene of *Rhodotorula glutinis* with irradiation and the assessment of key gene transcription. Bioresour. Technol..

[8] Braunwald T., Schwemmlein L., Graeff-Hönninger S., French W.T., Hernandez R., Holmes W.E., Claupein W. (2013). Effect of different C/N ratios on carotenoid and lipid production by *Rhodotorula glutinis*. Appl. Microbiol. Biotechnol..

[9] Nunes D.D., Pillay V.L., Van Rensburg E., Pott R.W.M. (2024). Oleaginous microorganisms as a sustainable oil source with a focus on downstream processing and cost-lowering production strategies: A review. Bioresour. Technol. Rep..

[10] Tang W., Wang Y., Zhang J., Cai Y., He Z. (2019). Biosynthetic pathway of carotenoids in *Rhodotorula* and strategies for enhanced their production. J. Microbiol. Biotechnol..

[11] Elfeky N., Elmahmoudy M., Zhang Y., Guo J.L., Bao Y. (2019). Lipid and carotenoid production by *Rhodotorula glutinis* with a combined cultivation mode of nitrogen, sulfur, and aluminium stress. Appl. Sci..

[12] Gong G., Liu L., Zhang X., Tan T. (2019). Multi-omics metabolism analysis on irradiation-induced oxidative stress to *Rhodotorula glutinis*. Appl. Microbiol. Biotechnol..

[13] Zhu Z., Zhang S., Liu H., Shen H., Lin X., Yang F., Zhou Y.J., Jin G., Ye M., Zou H. (2012). A multi-omic map of the lipid-producing yeast *Rhodosporidium toruloides*. Nat. Commun..

[14] Kimura K., Yamaoka M., Kamisaka Y. (2004). Rapid estimation of lipids in oleaginous fungi and yeasts using Nile red fluorescence. J. Microbiol. Methods.

[15] Sitepu R., Sestric R., Ignatia L., Levin D., German J., Gillies L.A., Almada L.A., Boundy-Mills K.L. (2013). Manipulation of culture conditions alters lipid content and fatty acid profiles of a wide variety of known and new oleaginous yeast species. Bioresour. Technol..

[16] Miller G.L. (1959). Use of Dinitrosalicylic Acid Reagent for Determination of Reducing Sugar. Anal. Chem..

[17] Mishra S., Suh W., Farooq W., Moon M., Shrivastav A., Park M.S., Yang J. (2014). Rapid quantification of microalgal lipids in aqueous medium by a simple colorimetric method. Bioresour. Technol..

[18] Van Wychen S., Ramirez K., Laurens L.M. (2013). Determination of Total Lipids as Fatty Acid Methyl Esters (FAME) by In Situ Transesterification.

[19] Frengova G., Sirnova E., Pavlova K., Beshkova D. (1994). Formation of Carotenoids by *Rhodotorula glutinis* in whey ultrafiltrate. Biotechnol. Bioeng..

[20] Weber R.W., Anke H., Davoli P. (2007). Simple method for the extraction and reversed-phase high-performance liquid chromatographic analysis of carotenoid pigments from red yeasts (Basidiomycota, Fungi). J. Chromatogr. A.

[21] Grabherr M.G., Haas B.J., Yassour M., Levin J.Z., Thompson D.A., Amit I., Adiconis X., Fan L., Raychowdhury R., Zeng Q. (2011). Full-length transcriptome assembly from RNA-Seq data without a reference genome. Nat. Biotechnol..

[22] Conesa A., Gotz S., Garcia-Gomez J.M., Terol J., Talon M., Robles M. (2005). Blast2GO: A universal tool for annotation, visualization and analysis in functional genomics research. Bioinformatics.

[23] Li B., Dewey C.N. (2011). RSEM: Accurate transcript quantification from RNA-seq data with or without a reference genome. BMC Bioinform..

[24] Saenge C., Cheirsilp B., Suksaroge T.T., Bourtoom T. (2011). Potential use of oleaginous red yeast *Rhodotorula glutinis* for the bioconversion of crude glycerol from biodiesel plant to lipids and carotenoids. Process Biochem..

[25] Wang Y., Zhang S., Zhu Z., Shen H., Lin X., Jin X., Jiao X., Zhao Z.K. (2018). Systems analysis of phosphate limitation-induced lipid accumulation by the oleaginous yeast *Rhodosporidium toruloides*. Biotechnol. Biofuels.

[26] Mondala A.H., Hernandez R., French T., McFarland L., Domingo J.W.S., Meckes M., Ryu H., Iker B. (2012). Enhanced Lipid and Biodiesel Production from Glucose-Fed Activated Sludge: Kinetics and Microbial Community Analysis. AIChE J..

[27] Dias C., Sousa S., Caldeira J., Reis A.T. (2015). New dual-stage pH control fed-batch cultivation strategy for the improvement of lipids and carotenoids production by the red yeast *Rhodosporidium toruloides* NCYC 921. Bioresour. Technol..

[28] El-Banna A.A., Abd El-Razek A.M., El-Mahdy A.R. (2012). Some Factors Affecting the Production of Carotenoids by *Rhodotorula glutinis* var. *glutinis*. Food Nutr. Sci..

[29] Bhosale P.B., Gadre R.V. (2001). Production of β-carotene by a mutant of *Rhodotorula glutinis*. Appl. Microbiol. Biotechnol..

[30] Han M., Xu Z.-Y., Du C., Qian H., Zhang W.-G. (2016). Effects of nitrogen on the lipid and carotenoid accumulation of oleaginous yeast *Sporidiobolus pararoseus*. Bioprocess Biosyst. Eng..

[31] Ratledge C. (2014). The role of malic enzyme as the provider of NADPH in oleaginous microorganisms: A reappraisal and unsolved problems. Biotechnol. Lett..

[32] Rashida Z., Srinivasan R., Cyanam M., Laxman S. (2021). Kog1/Raptor mediates metabolic rewiring during nutrient limitation by controlling SNF1/AMPK activity. Sci. Adv..

[33] Fraser P.D., Bramley P.M. (2004). The biosynthesis and nutritional uses of carotenoids. Prog. Lipid Res..

[34] Conrad M., Schothorst J., Kankipati H.N., Van Zeebroeck G., Rubio-Texeira M., Thevelein J.M. (2014). Nutrient sensing and signaling in the yeast Saccharomyces cerevisiae. FEMS Microbiol. Rev..

[35] Metur S.P., Klionsky D.J. (2024). Nutrient-dependent signaling pathways that control autophagy in yeast. FEBS Lett..

[36] Hariri H., Rogers S., Ugrankar R., Liu Y.L., Feathers J.R., Henne W.M. (2018). Lipid droplet biogenesis is spatially coordinated at ER–vacuole contacts under nutritional stress. EMBO Rep..

[37] Koh H.-J., Lee S.-M., Son B.-G., Lee S.-H., Ryoo Z.Y., Chang K.-T., Park J.-W., Park D.-C., Song B.J., Veech R.L. (2004). Cytosolic NADP+-dependent isocitrate dehydrogenase plays a key role in lipid metabolism. J. Biol. Chem..

[38] Lin Z., Ni C., Jiang H., Yang H., Deng L., Liu P., Li X., Yu Y., Li W., Wang R. (2025). Guanine Nucleotide Exchange Factors and Small GTPases: Their Regulation and Functions, Diseases, and Therapeutic Targets. MedComm.

[39] Yang Y., Ye Z., Guo M., Chen G. (2026). Transcriptomic analysis of the effects of nutritional conditions on *Rhodosporidium toruloides* lipid production. Biochem. Eng. J..

[40] Sasaki Y., Yoshikuni Y. (2022). Metabolic engineering for valorization of macroalgae biomass. Metab. Eng..

[41] Wu Y., Yan P., Li Y., Liu X., Wang Z., Chen T., Zhao X. (2020). Enhancing β-carotene production in *Escherichia coli* by perturbing central carbon metabolism and improving the NADPH supply. Front. Bioeng. Biotechnol..

[42] Hynes M.J., Murray S.L. (2010). ATP-citrate lyase is required for production of cytosolic acetyl coenzyme A and development in *Aspergillus nidulans*. Eukaryot. Cell.

[43] Partow S., Hyland P.B., Mahadevan R. (2017). Synthetic rescue couples NADPH generation to metabolite overproduction in Saccharomyces cerevisiae. Metab. Eng..

[44] Ratledge C. (2002). Regulation of lipid accumulation in oleaginous microorganisms. Biochem. Soc. Trans..

[45] Ratledge C., Wynn J.P. (2002). The biochemistry and molecular biology of lipid accumulation in oleaginous microorganisms. Adv. Appl. Microbiol..

[46] Dulermo T., Nicaud J.M. (2011). Involvement of the G3P shuttle and β-oxidation pathway in the control of TAG synthesis and lipid accumulation in *Yarrowia lipolytica*. Metab. Eng..

[47] Zhang S., Skerker J.M., Rutter C.D., Maurer M.J., Arkin A.P., Rao C.V. (2016). Engineering *Rhodosporidium toruloides* for increased lipid production. Biotechnol. Bioeng..

[48] Zhao X., Shi F., Zhan W. (2015). Overexpression of ZWF1 and POS5 improves carotenoid biosynthesis in recombinant Saccharomyces cerevisiae. Lett. Appl. Microbiol..

[49] Lee P.C., Momen A.Z.R., Mijts B.N., Schmidt-Dannert C. (2003). Biosynthesis of Structurally Novel Carotenoids in *Escherichia coli*. Chem. Biol..

[50] Choi S.K., Matsuda S., Hoshino T., Peng X., Misawa N. (2006). Characterization of bacterial β-carotene 3,3′-hydroxylases, CrtZ, and P450 in astaxanthin biosynthetic pathway and adonirubin production by gene combination in *Escherichia coli*. Appl. Microbiol. Biotechnol..

[51] Linden H. (1999). Carotenoid Hydroxylase from *Haematococcus pluvialis*: CDNA Sequence, Regulation and Functional Complementation. Biochim. Biophys. Acta.

[52] Mata-Gómez L.C., Montañez J.C.A., Méndez-Zavala A., Aguilar C.N. (2014). Biotechnological production of carotenoids by yeasts: An overview. Microb. Cell Fact..

